# Editorial: Education, training, and competency development for mental health-focused telehealth providers

**DOI:** 10.3389/fpsyg.2024.1443750

**Published:** 2024-06-18

**Authors:** Jonathan G. Perle, Wanhong Zheng, Lauren Gardner, Kari-Beth Law

**Affiliations:** ^1^Department of Behavioral Medicine and Psychiatry, Rockefeller Neuroscience Institute, West Virginia University School of Medicine, Morgantown, WV, United States; ^2^Center for Behavioral Health, Johns Hopkins All Children's Hospital, Saint Petersburg, FL, United States

**Keywords:** telehealth, telepsychology, telemedicine, telepsychiatry, digital health, competency, training, education

Telehealth is widely considered an umbrella term encompassing the integration of telecommunication technologies (e.g., video, telephone, email, messaging program) with healthcare services to support and promote clinical healthcare-focused prevention, assessment, intervention, consulting, research, training, supervision, data management advocacy, and professional communications. Technology becoming smaller, cheaper, more powerful, more interconnected, and more readily available has led telehealth to become ubiquitous with mental health practices (Perle, 2021). As with any novel healthcare service, an ethical, legal, research-informed, and safe telehealth practice requires the development of multiple knowledge- and applied skill-based competencies. Unfortunately, comprehensive activities to educate mental health providers in telehealth practices have remained relatively limited and fragmented, as has the evaluation of such programs. This has led to many citing variability in provider preparedness for ongoing telepractice (Glueckauf et al., 2018; Sammons et al., 2020; Baier and Danzo, 2021; Gardner et al., 2021; Perle, 2022), suggesting the need for both foundational and ongoing telehealth education, as well as real-world examples of effective implementation among providers to foster optimal care and service quality. In this Research Topic, readers can find a number of innovative articles designed to address mental health provider knowledge and education in telehealth-related practices across a variety of professional levels, locations, and methodologies.

In *Education and training of telemental health providers: a systematic review*, Jiang et al. consolidated literature between January 2013 and May 2023 from five electronically-indexed databases to extract telehealth training-pertinent information. As concentrated from an initial sample of 1,617, the final 20 articles were coded across variables of setting, target group, study aims, modality of training, quality metrics, and outcomes. The study demonstrated a high rate of variability among methods for training telehealth across professional level and location; however, combined to indicate the importance of telehealth training in improving measured knowledge, skills, and abilities among providers.

In *Preparing mental health providers for the future: the case for moving beyond the elective telehealth course to integrating telehealth training throughout the curriculum*, Wibberly highlighted the imperative need for comprehensive telehealth integration into graduate programming. Wibberly detailed a core competency framework for the integration of telehealth in the theoretical and practical facets of graduate education. This framework encompassed comprehensive understanding of technological platforms, ethics, legal and regulatory considerations, cultural competence, equity challenges, clinical assessment, and intervention approaches.

In *Evaluation of a multi-site health services psychology training program for telehealth and integrated behavioral health*, McCord et al. implemented experiential and didactic training in both telehealth and integrated behavioral health for health service psychology doctoral students within three partner organizations. The objectives of this training were to encourage remote warm hand-offs, hybrid shared appointments, therapy sessions, coordination with providers, supervision, consultation, mentorship, introduction to different parts of the healthcare system, and exposure to patient diversity. McCord et al. concluded multiple benefits of the training, including enhanced trainee confidence, autonomy, and independence; improved collaboration with other healthcare providers; greater adaptability; and increased openness to others' ideas. Despite benefits, provider readiness was an identified barrier to implementation across sites.

In *Bridging technology and care: integrating web-based PROMs in mental health services for refugees: a study on clinician training and technology adoption*, Moeller and Kring outlined the integration of web-based database technologies of patient-reported outcome measures (PROMs) with electronic health records within a specialized clinic located in Denmark for patients who were refugees with posttraumatic stress disorder. Overall, Moeller and Kring suggested positive provider experiences with the technology. Nevertheless, they further highlighted the importance of the alignment of technological systems with provider workflows, respect for professional judgement, and benefits to patient care for successful integration.

In *Virtual robotic telepresence early childhood mental health consultation to childcare centers in the aftermath of COVID-19: training approaches and perceived acceptability and usefulness*, Jent et al. explored a less-often studied area of telehealth utilization, childcare centers. Jent et al. examined how a multimedia/simulation training, and weekly communities of practice training influenced 10 early childhood consultants' use of a virtual robotic telepresence approach across 16 childcare centers in multilingual locations. These consultants subsequently trained 72 additional childcare staff members on the virtual methods. Findings indicated that consultants and childcare staff generally perceived the virtual telepresence consultation approach to be both useful and easy to implement. Additional hands-on training, as well as a hybrid approach for consultation were suggested by focus groups for future work.

In *Training, supervision, and experience of coaches offering digital guided self-help for mental health concerns*, Fitzsimmons-Craft et al. detailed the recruitment, rubric-based training, and supervision of 70 (37 completed post-intervention surveys) digital guided self-help coaches as part of a large randomized-controlled trial using digital platforms in order to meet the rising need for digital guided self-help programs for college students. Coaches were trained to provide digital guided self-help for depression, anxiety, and/or eating disorders via three live training sessions, training videos, and readings, followed by weekly supervision of their work. Overall, coaches suggested satisfaction with the training, as well as an increased ability to apply learned skills in their own practices for college students.

Finally, in *The COVID-19 pandemic, psychologists' professional quality of life and mental health*, Kercher et al. took a critical look into the COVID-19 pandemic and provider shifts to telehealth for 99 New Zealand-based psychologists via survey and questionnaires. The study targeted compassion fatigue, quality of life, depression, anxiety, stress, and resilience in psychologists. Combined information suggested a marked increase in work-related stress and compassion fatigue due to several factors, which included work and clinical demands, telehealth difficulties, and inadequate support. Taken together, Kercher et al. presented information to raise awareness for mental health provider burnout and stress during the COVID-19 pandemic in order to address future crises while also supporting mental health provider wellbeing.

As telehealth continues to evolve, it is important that research-informed methods for educating and training providers continue to be developed, evaluated, and utilized to guide ethical, legal, evidence-informed, and safe provider usage. Such studies can maximize outcomes, while reducing challenges that arise from the use of technology in clinical practice.

## Author contributions

JP: Writing – original draft, Writing – review & editing. WZ: Writing – original draft, Writing – review & editing. LG: Writing – original draft, Writing – review & editing. K-BL: Writing – original draft, Writing – review & editing.
